# A at Single Nucleotide Polymorphism-358 Is Required for G at -420 to Confer the Highest Plasma Resistin in the General Japanese Population

**DOI:** 10.1371/journal.pone.0009718

**Published:** 2010-03-16

**Authors:** Hiroshi Onuma, Yasuharu Tabara, Ryoichi Kawamura, Takashi Tanaka, Jun Ohashi, Wataru Nishida, Yasunori Takata, Masaaki Ochi, Kazuya Yamada, Ryuichi Kawamoto, Katsuhiko Kohara, Tetsuro Miki, Hideichi Makino, Haruhiko Osawa

**Affiliations:** 1 Department of Molecular and Genetic Medicine, Ehime University Graduate School of Medicine, Ehime, Japan; 2 Ehime Proteo-Medicine Research Center, Ehime University, Ehime, Japan; 3 Department of Basic Medical Research and Education, Ehime University Graduate School of Medicine, Ehime, Japan; 4 Laboratory of Molecular Biology, Faculty of Pharmacy, Osaka Ohtani University, Osaka, Japan; 5 Doctoral Program in Life System Medical Sciences, Graduate School of Comprehensive Human Sciences, University of Tsukuba, Ibaraki, Japan; 6 Department of Health and Nutritional Science, Faculty of Human Health Science, Matsumoto University, Nagano, Japan; 7 Department of Community Medicine, Ehime University Graduate School of Medicine, Ehime, Japan; 8 Department of Geriatric Medicine, Ehime University Graduate School of Medicine, Ehime, Japan; University of Utah, United States of America

## Abstract

Insulin resistance is a feature of type 2 diabetes. Resistin, secreted from adipocytes, causes insulin resistance in mice. We previously reported that the G/G genotype of single nucleotide polymorphism (SNP) at −420 (rs1862513) in the human resistin gene (*RETN*) increased susceptibility to type 2 diabetes by enhancing its promoter activity. Plasma resistin was highest in Japanese subjects with G/G genotype, followed by C/G, and C/C. In this study, we cross-sectionally analyzed plasma resistin and SNPs in the *RETN* region in 2,019 community-dwelling Japanese subjects. Plasma resistin was associated with SNP-638 (rs34861192), SNP-537 (rs34124816), SNP-420, SNP-358 (rs3219175), SNP+299 (rs3745367), and SNP+1263 (rs3745369) (*P*<10^−13^ in all cases). SNP-638, SNP -420, SNP-358, and SNP+157 were in the same linkage disequilibrium (LD) block. SNP-358 and SNP-638 were nearly in complete LD (*r^2^* = 0.98), and were tightly correlated with SNP-420 (*r^2^* = 0.50, and 0.51, respectively). The correlation between either SNP-358 (or SNP-638) or SNP-420 and plasma resistin appeared to be strong (risk alleles for high plasma resistin; A at SNP-358, *r^2^* = 0.5224, *P* = 4.94×10^−324^; G at SNP-420, *r^2^* = 0.2616, *P* = 1.71×10^−133^). In haplotypes determined by SNP-420 and SNP-358, the estimated frequencies for C-G, G-A, and G-G were 0.6700, 0.2005, and 0.1284, respectively, and C-A was rare (0.0011), suggesting that subjects with A at −358, generally had G at −420. This G-A haplotype conferred the highest plasma resistin (8.24 ng/ml difference/allele compared to C-G, *P*<0.0001). In THP-1 cells, the *RETN* promoter with the G-A haplotype showed the highest activity. Nuclear proteins specifically recognized one base difference at SNP-358, but not at SNP-638. Therefore, A at -358 is required for G at −420 to confer the highest plasma resistin in the general Japanese population. In Caucasians, the association between SNP-420 and plasma resistin is not strong, and A at −358 may not exist, suggesting that SNP-358 could explain this ethnic difference.

## Introduction

In mice, resistin is secreted from adipocytes, which antagonizes the action of insulin both *in vitro* and *in vivo* [1], [2]. While serum resistin levels are increased in obese diabetic mice, peroxisome proliferator–activated receptor γ (PPARγ) ligands reduce serum resistin [2]. Mice that overexpress the resistin gene (*retn*) in the liver are insulin-resistant through increased levels of serum resistin [3], while mice without *retn* show decreased fasting plasma glucose levels [4]. Therefore, elevated levels of serum resistin appear to be directly related to insulin resistance in mice. In humans, resistin appears to be mainly expressed in monocytes and macrophages [5], [6], [7].

Type 2 diabetes mellitus (T2DM) is characterized by insulin resistance in insulin target tissues, and impaired insulin secretion from pancreatic β cells [8]. It has been reported that some single nucleotide polymorphisms (SNPs), such as PPARγ Pro12Ala, KCNJ11 E23K, and TCF7L2 are associated with T2DM [9]. Recent genome-wide association studies (GWAS) have successfully identified ∼20 T2DM susceptibility SNPs, but the functional SNPs responsible for disease risk remain to be elucidated [10].

We previously carried out systematic analyses of SNPs in the human resistin gene (*RETN*), and found that the G/G genotype of a promoter SNP at −420 (rs1862513) was associated with T2DM susceptibility [11], [12]. Functionally, Sp1/3 transcription factors specifically recognized G at −420, resulting in enhanced *RETN* promoter activity. In the general Japanese population, subjects with the G/G genotype had the highest plasma resistin levels, followed by C/G and C/C [13]. However, the specific cis-acting SNPs that are responsible for determining circulating resistin levels around *RETN*, remains to be elucidated.

Melzer et al. reported that a SNP rs2431866, located at a distance of ∼100 Kb from *RETN*, is associated with circulating resistin in a genome-wide protein QTL (quantitative trait locus) analysis [14]. In an analysis of human lymphocyte samples from 1,240 adults, Tejero et al. reported that a QTL that influences *RETN* mRNA is localized on chromosome 19p, where the strongest positional candidate is *RETN* itself [15]. In a previous study, we reported that monocyte *RETN* mRNA was positively correlated with its simultaneous serum level [16]. To our knowledge, no cis-acting SNPs have been shown to have stronger effects on their relevant gene products than SNP-420. Therefore, circulating resistin, as a model of protein QTL, merits further investigation, and SNPs in the vicinity of *RETN* would be of special interest.

In view of this, to determine the SNPs responsible for circulating resistin around *RETN*, we cross-sectionally analyzed 2,019 Japanese subjects. Plasma resistin was found to be most strongly associated with SNP-358 and SNP-420, where the relevant allele, A at-358, was tightly linked to G at −420.

## Results

### SNP-358, and SNP-638 were in nearly complete linkage disequilibrium (LD), and were tightly correlated with SNP-420 in the general Japanese population

**Figure 1:**
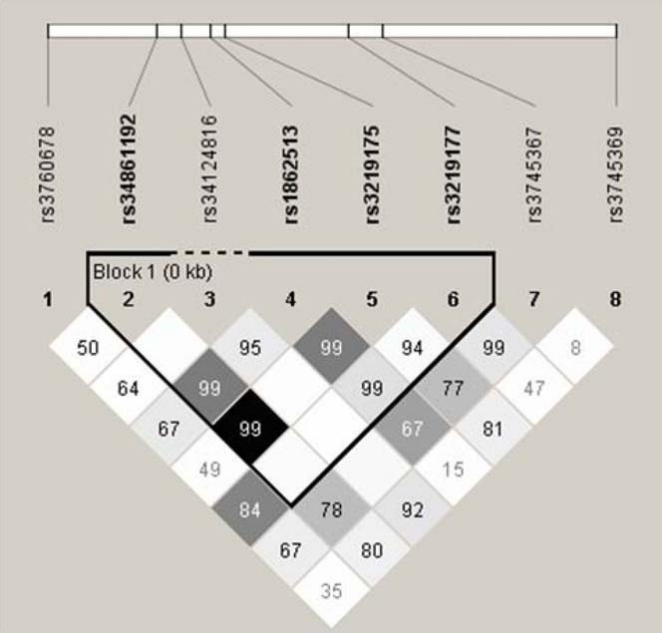
SNP-358, and SNP-638 were nearly in complete LD, and were tightly correlated with SNP-420 in the general Japanese population. The 8 SNPs around *RETN* were genotyped in 2,019 subjects in the general Japanese population, and LD between each pair of these SNPs were analyzed by Haploview [28]. One LD block consisting of SNP-638, SNP -420, SNP-358, and SNP+157 was defined by the confidence interval analysis. Each square represents a pairwise value of *D*', with the standard gradation (black indicates LOD> = 2 and *D*' = 1; none indicates, LOD<2 and *D*' = 1; white indicates LOD<2 and D' <1, gray indicates LOD> = 2 and *D*'<1). The *r^2^* between SNP-638 and SNP-358 was 0.98. The *r^2^* between SNP-420 and SNP-358 or SNP-638 was 0.50, and 0.51, respectively.

We first typed the 8 SNPs listed in Table 1. All of these SNPs were considered to be in Hardy-Weinberg equilibrium because *P* = 0.049 for SNP+157, and *P* = 0.037 for SNP+1263 did not remain significant after Bonferroni's correction. Based on a confidence interval analysis, SNP-638, SNP -420, SNP-358, and SNP+157 were determined to be in the same LD block (Fig. 1). Four major haplotypes defined by SNP-638, SNP-420, SNP-358, and SNP+157 were found (haplotype, frequency; G-C-G-C, 0.670; A-G-A-C, 0.200; G-G-G-C, 0.073; G-G-G-T, 0.055). Approximately 94.5% of chromosomes can be captured by two tag SNPs, namely, SNP-358 (or SNP-638) and SNP-420. SNP-358 and SNP-638 were in nearly complete LD (*r^2^* = 0.98), and were tightly correlated with SNP-420 (*r^2^* = 0.50, and 0.51, respectively).

**Table 1:** Association between each SNP around *RETN* and plasma resistin in the general Japanese population.

SNP (rs No.)	Genotype	*n*	HWE	Plasma resistin (ng/ml)			
				mean **±** SD	*F*	*r^2^*	*P*
−1093A>G	A/A	1705	0.310	11.5±6.5	0.07	7.4*10^−5^	0.929
(rs3760678)	A/G	297		11.6±7.3			
	G/G	17		11.6±4.0			
−638G>A	G/G	1304	0.056	8.2±3.3	1125.489	0.5275	2.75×10^−329^*
(rs34861192)	G/A	620		16.5±5.9			
	A/A	95		24.5±7.6			
−537A>C	A/A	1855	0.417	11.8±6.7	31.18	0.0300	4.63×10^−14^*
(rs34124816)	A/C	159		7.8±3.9			
	C/C	5		5.2±3.0			
−420C>G	C/C	921	0.241	8.1±3.2	357.13	0.2616	1.71×10^−133^*
(rs1862513)	C/G	868		13.3±6.5			
	G/G	230		18.0±9.1			
−358G>A	G/G	1300	0.073	8.2±3.3	1102.52	0.5224	4.94×10^−324^*
(rs3219175)	G/A	624		16.4±5.9			
	A/A	95		24.5±7.7			
+157C>T	C/C	1804	0.049	11.6± 6.6	1.62	0.0016	0.198
(rs3219177)	C/T	204		10.8±6.5			
	T/T	11		10.1±3.6			
+299G>A	G/G	796	0.359	8.5±3.9	214.26	0.1753	5.24×10^−85^*
(rs3745367)	G/A	928		12.4±6.6			
	A/A	295		16.5±8.2			
+1263G>C	G/G	1074	0.037	12.6±7.2	36.64	0.0351	2.33×10^−16^*
(rs3745369)	G/C	822		10.4±5.7			
	C/C	123		9.2±4.4			

ANOVA is used for statistical analysis (*n* = 2,019). HWE, Hardy-Weinberg Equilibrium. *, these *P* values remained significant after Bonferroni's correction (raw *P* value ×8).

### Nuclear proteins specifically recognized a difference in one base at SNP-358 but not at SNP-638

**Figure 2:**
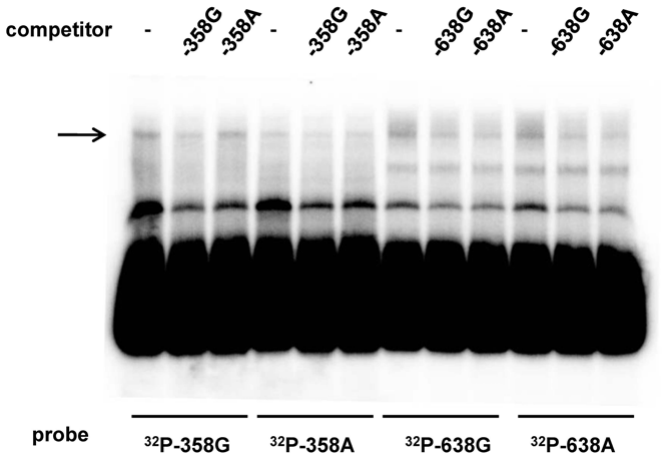
Nuclear proteins specifically recognized a difference in one base at SNP-358 but not at SNP-638. EMSA was performed as described in *Methods*. A labeled probe representing *RETN* promoter sequences around SNP-358 contained G (major allele) or A (minor allele) at SNP-358 (**^32^**P-358G or **^32^**P-358A, respectively), and that around SNP-638 contained G (major allele) or A (minor allele) at SNP-638 (**^32^**P-638G or **^32^**P-638A, respectively). Each probe was incubated with a nuclear extract of THP-1 cells in the absence (−) or presence of a 200-fold molar excess of unlabeled competitor double-stranded oligonucleotides indicated (−358G, −358A, −638G, or −638A). The arrow points to the band that specifically bound to the probe with G at SNP-358.

To determine which SNP, i.e., SNP-358 or SNP-638, is potentially functional, we examined whether nuclear proteins specifically bound to DNA sequences using electrophoretic mobility shift assay (EMSA) (Fig. 2). Nuclear proteins were found to bind to a labeled probe with G at SNP-358 (-358G probe)(1st lane from the left), which was reduced by a cold competitor with G at SNP-358, but not by that with A at SNP-358 (2nd and 3rd lanes, respectively). This suggests that nuclear proteins specifically recognized a difference in one base at SNP-358. Consistent with this, no specific proteins bound to a labeled probe with A at SNP-358 (4th to 6th lanes). Regarding SNP-638, non-specific protein binding was found with a −638G probe (lane 7th), which was evenly reduced by a cold competitor with G at −628 (8th lane) and that with A at −638 (9th lane). The same non-specific protein binding was observed for a −638A probe (10th to 12th lanes). In addition, a computer search (MatInspector program (http://www.genomatix.de/)) revealed the presence of transcription factor binding sites for SNP-358 but not for SNP-638. Therefore, we tentatively concluded that SNP-358 was a more functional SNP than SNP-638, and used SNP-358 when necessary in this study.

### Plasma resistin was associated with SNP-638, SNP-537, SNP-420, SNP-358, SNP+299, and SNP+1263 in the general Japanese population

We next examined the relation between plasma resistin levels and each of the 8SNPs (Table 1). Plasma resistin levels were associated with SNP-638 (*P* = 2.75×10^−329^), SNP-537 (*P* = 4.63×10^−14^), SNP-420 (*P* = 1.71×10^−133^), SNP-358 (*P* = 4.94×10^−324^), SNP+299 (*P* = 5.24×10^−85^), and SNP+1263 (*P* = 2.33×10^−16^). These *P* values remained significant after Bonferroni's correction. A low *P* value and high *r^2^*were found for SNP-358 (or SNP-638), and SNP-420 (*r^2^* = 0.5224 (0.5275), and 0.2616, respectively). The difference in plasma resistin levels between each genotype appears to be highest for SNP-358 (or SNP-638), followed by SNP-420 (∼8 ng/ml for SNP-358, and ∼5 ng/ml for SNP-420). These findings remained unchanged when assessed using multiple regression analyses adjusted for age, gender, and BMI (Table 2).

**Table 2:** Multiple regression analysis involving plasma resistin (ng/ml) as a dependent variable and each SNP as an independent variable (adjusted for age, gender, and BMI).

SNP	Reference	Independent variable	Unstandardized regression coefficient	Standard error	Standardized regression coefficient	*P*
−1093A>G (rs3760678)	AA	A/G	0.17	0.41	0.01	0.688
		G/G	0.38	1.60	0.01	0.813
−638G>A (rs34861192)	GG	G/A	8.31	0.22	0.58	2.19×10^−240^
		A/A	16.43	0.47	0.53	7.66×10^−207^
−537A>C (rs34124816)	AA	A/C	−4.11	0.53	−0.17	1.82×10^−14^
		C/C	−7.09	2,89	−0.05	0.014
−420C>G (rs1862513)	CC	C/G	5.16	0.27	0.39	3.08×10^−77^
		G/G	9.85	0.41	0.47	1.08×10^−110^
−358G>A (rs3219175)	GG	G/A	8.25	0.22	0.58	1.80×10^−236^
		A/A	16.40	0.48	0.53	1.55×10^−204^
+157C>T (rs3219177)	CC	C/T	0.40	2.02	0.02	0.843
		T/T	1.14	1.98	0.05	0.565
+299G>A (rs3745367)	GG	G/A	3.93	0.29	0.30	5.05×10^−41^
		A/A	8.05	0.40	0.43	8.97×10^−81^
+1263G>C (rs3745369)	GG	G/C	−2.22	0.30	−0.17	1.43×10^−13^
		C/C	−3.57	0.61	−0.13	6.05×10^−9^

Multiple regression analyses involving plasma resistin (ng/ml) as a dependent variable and each SNP genotype, age (years), gender (male = 0, female = 1), and BMI as independent variables were performed as described in Methods. For each SNP genotype, S/S was compared to N/N, and N/S was compared to N/N, respectively where the major and minor alleles based on the allele frequency were defined by normal (N), and susceptibility (S) alleles, respectively.

When the combination of SNP-420 and SNP-358 genotypes was analyzed, all subjects having the A/A genotype of SNP-358 also had the G/G genotype of SNP-420, and showed the highest plasma resistin levels (ANOVA, *P*<0.001, and Scheffe, *P*<0.001 compared to subjects with each of the other genotypes)(Table 3). Since only 4 of the 921 subjects with the C/C genotype of SNP-420 appeared to be heterozygotes of A at-358, and none was this homozygote, it was not possible to conclude the existence of an effect of A at-358, independent of G at −420. It is noteworthy that findings reported in two previous studies suggest that the effect of G at −420 is independent of A at SNP-358 *in vitro* [12], [17]. Therefore, the G of SNP-420 and the A of SNP-358 are both possible primary SNPs with the potential function of determining high plasma resistin levels around *RETN* in the general Japanese population.

**Table 3:** Plasma resistin in subjects with each combination of *RETN* SNP-420, and SNP-358 genotypes.

SNP-420	SNP-358		
	G/G	G/A	A/A
C/C	8.1±3.1 (917)	10.6±6.3 (4)	N/A (0)
C/G	8.3±3.8 (339)	16.6±5.7 (529)	N/A (0)
G/G	7.9±3.1 (44)	16.0±6.8 (91)	24.5±7.7 (95)

Plasma resistin (mean±SD) (ng/ml) is shown for each combination of SNP-420, and SNP-358 genotypes. The number of subjects is shown in parenthesis. Plasma resistin was higher in subjects with SNP-420G/G and SNP-358A/A genotypes than those with any other genotypes (ANOVA, *P*<0.001; Scheffe, *P*<0.001 compared to each of the other genotypes). N/A, not applicable because no subjects had this combination.

### The G-A haplotype defined by SNP-420 and SNP-358 conferred the highest plasma resistin in the general Japanese population

To determine which combination of these potentially functional SNPs is most responsible for plasma resistin, we examined the relation between plasma resistin and haplotypes defined by SNP-420 and SNP-358 in this order (Table 4). The estimated frequencies for C-G, G-A, and G-G were 0.6700, 0.2005, and 0.1284, respectively. Since the C-A haplotype was rare (0.0011), subjects having A at −358, generally had a G at −420. Conversely, ∼60% of chromosomes having G at −420 had A at −358. The highest risk haplotype, the G-A haplotype, conferred an 8.24 ng/ml higher plasma resistin level than a low risk reference haplotype, C-G (P<0.0001). Although C-A was estimated to show a 2.47 ng/ml higher plasma resistin level, compared to C-G (*P* = 0.2700), it was not possible to verify an effect of A at −358 itself, independent of G at −420, due to its low frequency (0.0011). The G-G haplotype, with G at −420, but not A at −358, showed an effect similar to that for C-G (−0.05 ng/ml difference, *P* = 0.8000). Therefore, G at −420 was not sufficient to confer the highest plasma resistin, and A at −358 was required in the general Japanese population. It is difficult to determine whether A at −358 alone or in combination with G at −420 is more responsible for this effect, based on only statistical analyses of genetic data.

**Table 4:** Estimated frequency and plasma resistin in each haplotype defined by *RETN* SNP-420, and SNP-358 genotypes.

SNP-420	SNP-358	Estimated frequency	Difference of plasma resistin (95% *CI*) (ng/ml)	*P*
C	G	0.6700	0.00 = reference	–
G	A	0.2005	8.24(7.89–8.59)	<0.0001
G	G	0.1284	−0.05(−0.47–0.37)	0.8000
C	A	0.0011	2.47 (−1.89–6.83)	0.2700

Frequency and plasma resistin for each haplotype defined by *RETN* SNP-420 and SNP-358 genotypes were estimated by SNPstats [29]. Estimated differences of plasma resistin compared to a reference haplotype, C-G, are shown in each haplotype.

### The *RETN* promoter with the G-A haplotype defined by SNP-420 and SNP-358 showed the highest activity in THP-1 cells

**Figure 3:**
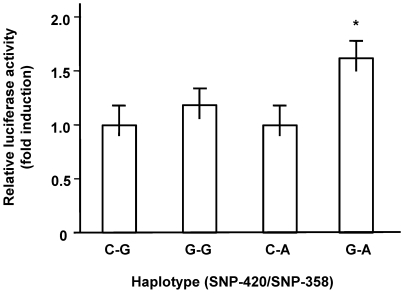
The *RETN* promoter with the G-A haplotype defined by SNP-420 and SNP-358 showed the highest activity in THP-1 cells. The *RETN* promoter reporter including −450/206 of *RETN* with each haplotype defined by SNP-420 and SNP-358 was transiently transfected into THP-1 human monocytes. Luciferase activity was measured as described in Methods. Relative luciferase activities are shown as the mean fold induction ± SE relative to the activity of the reference promoter reporter with the C-G haplotype. The data represent eight independent experiments with triplicate wells for each condition. ANOVA, *P* = 0.002. *, Scheffe, *P* = 0.008 compared to C-G. The base numbers were shown where the translation initiation site was defined as +1.

Finally, to determine the functional relevance of haplotypes defined by SNP-420 and SNP-358, we performed transfection experiments using THP-1 human monocytes. The *RETN* promoter activity with G at −420 and A at−358 showed the higher activity than the reference control promoter with C at −420 and G at−358 (*n* = 8, mean ± SE, 1.62±0.16 fold; ANOVA , *P* = 0.002; Scheffe, *P* = 0.008) (Fig. 3). The promoter activity with the other two haplotypes, namely, G-G and C-A was not different from that with C-G. Since SNP-638 was not included in these promoter constructs, the observed effect was independent of A at SNP-638. Therefore, from statistical analyses of genetic data of the general Japanese population, and *in vitro* functional data, A at SNP-358 is required for G at -420 to confer the highest plasma resistin in the general Japanese population.

## Discussion

These findings herein indicate that plasma resistin levels are associated with SNP-638, SNP-537, SNP-420, SNP-358, SNP+299, and SNP+1263 around *RETN* in the general Japanese population. The lowest *P* value was observed for SNP-358 (or SNP-638), followed by SNP-420. SNP-420, and SNP-358 were in the same LD block, and a risk allele, A of SNP-358, was tightly linked with a risk allele, G of SNP-420. The G-A haplotype defined by SNP-420, and SNP-358, which included these risk alleles, conferred the highest plasma resistin. Functionally, the *RETN* promoter with this haplotype showed the highest activity in THP-1 cells. Nuclear proteins specifically recognized a difference in one base at SNP-358, but not at SNP-638.

We found that SNP-358 was associated with plasma resistin in the order of A/A >G/A>G/G in a large number of Japanese subjects. Subjects having the relevant allele for high plasma resistin, i.e. an A at−358, generally had a G at −420. In four independent studies, the activity of the mutant *RETN* promoter including G at −420 was higher than that of the wild type including C at −420 *in vitro* [12], [17], [18], [19]. Of these, two studies have suggested that the effect of G at −420 is independent of A at SNP-358. In a previous study, we showed that Sp1/3 enhanced *RETN* promoter activity with G at −420 and G at−358 in the absence of SNP-638 in SL2 cells [12]. The findings reported herein indicate that *RETN* promoter activity with G at −420 and A at−358 without SNP-638 was ∼60% higher than that with the other combinations. Therefore, both the A of SNP-358 and the G of SNP-420 appear to be required for the highest *RETN* promoter activity.

During the preparation of this manuscript, Asano et al. reported on-line that rs34861192 (SNP-638G>A) and rs3745268 (3′ UTR) are significant determinants of plasma resistin in an aged Japanese population [20]. Whereas their SNP typing data were similar to our results, the analysis and interpretation were different from those reported here. Based exclusively on statistical analyses of genetic data, they concluded that SNP-638 was a more promising candidate than SNP-420. However, SNP-638, SNP-420, and SNP-358 were all located in the same LD block, and all of these three SNPs were strongly associated with circulating resistin levels. It should be pointed out that it is difficult to identify a causal variant from highly correlated SNPs by the exclusive use of statistical analyses of genetic data without other information [21]. Therefore, a haplotype analysis and functional data were taken into account in assessing promising causal variants in the present study.

Since the A at −358 was tightly linked with the G at−420, the issue of whether the A of SNP-358 itself is largely responsible for high plasma resistin levels, or whether both the A of SNP-358 and the G of SNP-420 are required remains to be elucidated. Also, the possible relevance of SNP-358 is suggested, since the sequences around SNP-358 are similar to the NF-κB consensus sequences based on the MatInspector program (http://www.genomatix.de/). The induction of *RETN* expression by lipopolysaccharides is inhibited by an NF-κB inhibitor in human macrophages [22]. Although no transcription factor binding sites were detected around SNP-638 by this program, it should be noted that SNP-638 had the same statistical association as SNP-358. The lack of apparent nuclear protein binding to the DNA sequence around SNP-638 may have resulted from inappropriate reaction conditions in detecting specific binding in the present study. SNP-638, and other SNPs including SNP+299 which show low *P* values clearly merit further investigation.

We previously reported that a functional promoter SNP-420 was tightly associated with plasma resistin, its final gene product, in Japanese subjects [12], [13], [16]. This association is also supported by a study reported by Cho et al. in an analysis of Korean subjects [18], but not that by Menzaghi et al. in an analysis of Caucasian subjects [23]. Most recently, Hivert et al. also reported that SNP-420 was not associated with circulating resistin in Caucasian subjects [24]. In their meta-analysis, which included Caucasians, Japanese, and Korean subjects, SNP-420 was found to be associated with plasma resistin with significant heterogeneity among the groups studied, suggesting that ethnic differences may be a factor [24]. In fact, whereas the A allele of SNP-358, which is tightly linked with the G allele of SNP-420, showed the strongest association with high plasma resistin in the present study, the A allele of SNP-358 was not found in the HapMap CEU population (http://www.hapmap.org/index.html.en). Therefore, SNP-358 is likely to be monophormic in Caucasians, which could account for the ethnic differences in the association between plasma resistin and SNP-420.

Most recently, in “humanized resistin mice”, where human resistin is specifically expressed in macrophages and endogenous *retn* is disrupted, a high fat diet was found to induce insulin resistance with inflammation of adipose tissue [25]. Although this supports the pathophysiological relevance of human resistin in insulin resistance, it remains controversial whether circulating resistin is associated with insulin resistance, T2DM, or adiposity in humans [1], [13]. The discrepancy among previous reports may be resolved by analyzing SNPs that are correlated with circulating resistin. The lower power with a smaller numbers of subjects may also explain this difference. The broader range of the assay used in this study could also be a contributing factor. In addition, serum resistin probably exists as a hexamer (major form) or trimer (a more biologically active form) in mice [26]. The presence of multimers in human serum has been suggested, and this may also have affected the assay results [27].

In summary, of the potentially functional SNPs, SNP-358 appears to be most strongly associated with plasma resistin in the general Japanese population, followed by SNP-420. Subjects having the responsible allele for the highest plasma resistin, A at −358, generally had G at −420. The G-A haplotype defined by SNP-420 and SNP-358 conferred the highest plasma resistin. Functionally, the *RETN* promoter with this G-A haplotype showed the highest activity. It is not clear why A of SNP-358 is required for G of SNP-420 to confer the highest plasma resistin, and whether the A of SNP-358 indeed accounts for ethnic differences in the association between plasma resistin and SNP-420. Further studies will be required to clarify these points.

## Methods

### Ethics Statement

All subjects were informed of the purpose of the study and their written consent was obtained. The study was approved by the ethics committee of the Ehime University Graduate School of Medicine.

### Subjects

This cross-sectional study included community-dwelling Japanese subjects who were attending an annual medical check-up in a rural town located in Ehime prefecture in Japan. We previously analyzed resistin SNP-420 and plasma resistin levels in 2,078 subjects in this population [13]. Of these subjects, 2,019 subjects, in whom all of the eight SNPs examined in the present study were successfully typed, were analyzed. The baseline characteristics of the study subjects, such as alcohol habituation, history or symptoms of cardiovascular disease (CVD), and medication-taking were investigated by an individual interview using a structured questionnaire. The clinical characteristics of these subjects are summarized in Table 5.

**Table 5:** Characteristics of the population studied (*n* = 2,019).

Sex (male/female)	883/1136
Age (years)	62.1±12.6
Body mass index (kg/m^2^)	23.4±3.2
Systolic blood pressure (mmHg)	138.8±22.4
Diastolic blood pressure (mmHg)	81.9±11.9
Total cholesterol (mg/dl)	203.4±34.7
Glucose (mg/dl)	98.4±21.7
Insulin (µU/ml)	6.8±5.0
HOMA-IR	1.6±1.4
Resistin (ng/ml)	11.5±6.6
Current smoking (%)	27.8
Current drinking (%)	57.2
History of cardio vascular diseases (%)	7.3
Medication (%)	
Hypertension	25.9
Type 2 diabetes	3.4
Hyperlipidemia	5.8

Values are means±SD, or *n* (%). Number of subjects who measured immunoreactive insulin was 1,958. HOMA-IR (homeostasis model assessment insulin resistance index) was calculated as glucose (mg/dl) x insulin (µU/ml)/405. CVD indicates cardiovascular disease, including stroke, myocardial infarction, and angina pectoris.

### SNP typing

The seven SNPs were selected, based on our systemic analysis of SNPs in *RETN* [11], [12], and one SNP (rs3745369) in the 3′ flanking region was added from HapMap (http://www.hapmap.org/index.html.en). These SNPs included all of the 4SNPs reported in HapMap around *RETN*. SNPs were typed by TaqMan analysis using assay on demand probes and primers sets (Applied Biosystems) except rs3760678, rs34861192, rs34124816, and rs3219177 which were generated as assay by design. The rs1862513 was previously typed as described [13]. Genotype call rates were rs3760678 (99.2%), rs34861192 (99.5%), rs34124816 (98.4%), rs3219175 (99.3%), rs3219177 (99.3%), rs3745367 (99.3%), and rs3745369 (99.7%).

### Measurement of plasma resistin

Fasting plasma resistin was determined using a human resistin ELISA kit (LINCO Research Inc., St. Charles, MO), according to the manufacturer's protocol [12]. The assay was linear below 0.16 ng/ml. Inter- and intra-assay coefficients of variation (CVs) were 6.9 and 1.7% (low levels), and 7.2 and 8.1% (high levels), respectively.

### Electrophoretic mobility shift assay (EMSA)

THP-1 cells were grown in RPM1 medium supplemented with 10% fetal bovine serum at 37°C in a 5% CO_2_ incubator. Nuclear extracts of THP-1 cells were prepared with NE-PER^®^ Nuclear and Cytoplasmic Extraction Reagents (Thermo SCIENTIFIC). The following oligonucleotides were used for EMSA as probes or competitors, sense hRE-358A, 5′-CACTGTCTGCTCAGGAGCTTCCTCTTGGCCA-3′; antisense hRE-358A, 5′-GATCTGGCCAAGAGGAAGCTCCTGAGCAGACAGTGGTAC-3′; sense hRE-358G, 5′-CACTGTCTGCTCAGGGGCTTCCTCTTGGCCA-3′; antisense hRE-358G, 5′-GATCTGGCCAAGAGGAAGCCCCTGAGCAGACAGTGGTAC-3′, sense hRE-638A, 5′-CGTCACTGTAGCTTCAAACTCCCGGGCTCAA-3′; antisense hRE-638A, 5′-GATCTTGAGCCCGGGAGTTTGAAGCTACAGTGACGGTAC-3′; sense hRE-638G, 5′-CGTCACTGTAGCTTCGAACTCCCGGGCTCAA-3′; antisense hRE-638G, 5′-GATCTTGAGCCCGGGAGTTCGAAGCTACAGTGACGGTAC-3′. Complementary oligonucleotides were annealed and labeled with ^32^P. A 10 µg of nuclear extract was incubated for 30 min with a ^32^P-labeled oligonucleotide (0.01 pmol) and 1 µg of poly(dAdT) in reaction buffer containing 25 mM HEPES (pH 7.9), 0.5 mM EDTA, 0.5 mM DTT, 40 mM KCl and 4% Ficoll (v/v). Nonidet P-40 (0.5%), and a 100-fold molar excess of the unlabeled single-stranded sense and antisense oligonucleotides, corresponding to each labeled probe were added to reduce nonspecific binding. Competition experiments were performed by the addition of a 200-fold molar excess of unlabelled double-stranded oligonucleotides to the reaction mixture. After the binding reaction, the mixture was subjected to 6% PAGE in 45 mM Tris, 45 mM boric acid, 1 mM EDTA at 100V for 2 h at 4°C. The gel was dried and exposed to a FUJIFILM imaging plate. Signals were detected with the FUJIFILM FLA-5100 image analyzing system.

### Plasmid construction

The 5′ flanking region between −450 and −206 of *RETN* was amplified by PCR using DNA from either a subject homozygous for a wild-type allele (−420C) or a subject homozygous for a mutant allele (−420G). These products were inserted into pGL3 basic vector (Promega, Madison, WI) to generate pGL3(−420G) and pGL3(−420C), both of which harbor G at the −358 locus, as described previously[12]. These constructs were modified by using QuickChange II Site-Directed Mutagenesis Kit (STRATAGENE, La Jolla, CA) to generate a point mutation at −358 from G to A. The primers used were 5′-CTTACTGTCTGCTCAGGAGCTTCCTCTTGGCCCCG, and 5′- CGGGGCCAAGAGGAAGCTCCTGAGCAGACAGTAAG. The DNA fragments between −450 and −206 of *RETN* with or without this point mutation were subsequently cloned into a luciferase reporter vector pGL4.11 (Promega) at the site between *Kpn I* and *Bgl II*. The sequence of the generated four plasmids, pGL4(−420C/−358G), pGL4(−420C/−358A), pGL4(−420G/−358G) and pGL4(−420G/−358A), were confirmed by DNA sequencing. All plasmids used for the transfection were prepared by use of a Qiagen plasmid kit, followed by CsCl_2_ density-gradient ultracentrifugation as described previously [12].

### Transient transfection and luciferase assay

THP-1 human monocytes purchased from Human Science Research Resources Bank (Osaka, Japan) were grown in RPMI1640 containing 10% FCS. One day before transfection, THP-1 cells were plated on a 6 well plate at 4.0×10^5^ cells/well. Trans IT-Jurkat Reagent (Mirus, Madison, WI) was used for transient transfection following the manufacturer's protocol. Each of the pGL4(−420C/−358G), pGL4(−420C/−358A), pGL4(−420G/−358G) and pGL4(−420G/−358A) vectors was transfected into these cells along with pGL4.11 basic vector (control for basal activity), pGL4.11 promoter vector (positive control), and pRL-SV40 internal control vector. The THP-1 cells were then incubated with RPMI1640 containing 10% FCS for 48 hours. Luciferase assay were performed using the Dual-Luciferase^TM^ Reporter Assay System according to the manufacturer's protocol (Promega).

### Statistical analysis

In the human population study, values are expressed as the mean±standard deviation. Differences in plasma resistin levels among genotypes were assessed by analysis of variance (ANOVA). Bonferroni's correction was applied to Table 1 (raw *P* value ×8). To assess isolated effects of each SNP on plasma resistin, multiple regression analyses involving each SNP, age (year), gender (male = 1, female = 0), and BMI as independent variables, and plasma resistin (ng/ml) as a dependent variable were performed. In these analyses, we assume that the major and minor alleles based on the allele frequency are normal (N), and susceptibility (S) alleles, respectively. Each SNP genotype, N/N, N/S, and S/S, were denoted by two dummy variables (c1, c2)  = (0, 0), (1, 0), and (0, 1), respectively. This first dummy variable estimates the difference between N/S and N/N, and the second between S/S and N/N. These analyses were performed using a commercially available statistical software package (JMP ver. 7, SAS Institute, Cary, NC). Linkage disequilibrium (LD) was assessed using the Haploview software [28]. Frequency and plasma resistin in each haplotype defined by SNP-420 and SNP-358 genotypes were estimated using SNPstats [29]. ANOVA followed by Scheffe as a post-hoc test was used where indicated. Null hypotheses were rejected at *P*<0.05.
