# Correction: A at Single Nucleotide Polymorphism-358 Is Required for G at -420 to Confer the Highest Plasma Resistin in the General Japanese Population

**DOI:** 10.1371/annotation/7b2a49da-b02f-4185-8eaf-3a30ad7f5b89

**Published:** 2010-03-26

**Authors:** Hiroshi Onuma, Yasuharu Tabara, Ryoichi Kawamura, Takashi Tanaka, Jun Ohashi, Wataru Nishida, Yasunori Takata, Masaaki Ochi, Kazuya Yamada, Ryuichi Kawamoto, Katsuhiko Kohara, Tetsuro Miki, Hideichi Makino, Haruhiko Osawa

An error in the author byline has been corrected. The new citation is: Onuma H, Tabara Y, Kawamura R, Tanaka T, Ohashi J, et al. (2010) A at Single Nucleotide Polymorphism-358 Is Required for G at -420 to Confer the Highest Plasma Resistin in the General Japanese Population. PLoS ONE 5(3): e9718. doi:10.1371/journal.pone.0009718.

